# Circular RNA Expression and Interaction Patterns Are Perturbed in Amyotrophic Lateral Sclerosis

**DOI:** 10.3390/ijms232314665

**Published:** 2022-11-24

**Authors:** Chiara Aquilina-Reid, Samuel Brennan, Ashton Curry-Hyde, Guus M. Teunisse, The NYGC ALS Consortium, Michael Janitz

**Affiliations:** 1 GenieUs Genomics Pty Ltd., Sydney, NSW 2010, Australia; 2 School of Biotechnology and Biomolecular Sciences, University of New South Wales, Sydney, NSW 2052, Australia; 3 The New York Genome Center, 101 Avenue of the Americas, New York, NY 10013, USA; 4 Paul Flechsig Institute for Brain Research, University of Leipzig, 04103 Leipzig, Germany

**Keywords:** amyotrophic lateral sclerosis, circular RNAs, microRNAs, spinal cord, transcriptome sequencing

## Abstract

Circular RNAs (circRNAs) are a type of long noncoding RNA that are highly abundant and highly conserved throughout evolution and exhibit differential expression patterns in various tissue types in multiple diseases, including amyotrophic lateral sclerosis (ALS). The most well-known function of circRNAs is their ability to act as microRNA (miRNA) sponges. This entails circRNA binding to miRNA, which would otherwise target and degrade messenger RNA, thus affecting gene expression. This study analyzed ALS patient samples from three spinal cord regions to investigate circular transcriptome perturbation and circular RNA–microRNA–mRNA interactions. Using stringent statistical parameters, we identified 92 differentially expressed circRNAs across the spinal cord tissues with the aim of identifying specific circRNAs with biomarker potential. We also found evidence for differential expression of 37 linear RNAs possibly due to miRNA sequestration by circRNAs, thus revealing their potential as novel biomarkers and therapeutic candidates for ALS.

## 1. Introduction

Amyotrophic lateral sclerosis (ALS) is a progressive, neurodegenerative disease that correlates with multiple pathogenic mechanisms as well as a wide range of clinical manifestations and markers of disease progression [1]. ALS has a low median incidence rate of 5.4 cases per 100,000 in people aged between 58 and 65 [2,3]; however, the very short life expectancy of 30 months on average from the onset of the first symptoms confers a dismal prognosis [1]. The symptoms observed are due to degeneration of motor neurons in the motor cortex and spinal cord and may consist of muscle weakness, cramping and twitching, progressing to muscle atrophy [4].

There are two main types of ALS: familial ALS, in which the patient inherits disease-causing mutations, and sporadic ALS, in which no family history of the disease or primary genetic cause can be identified. The mechanisms by which ALS begins and progresses are unclear, rendering patient diagnosis difficult and determining viable treatment options complex. Multiple molecular mechanisms are involved in ALS pathogenesis; however, there is no distinctive biological pathway to date.

Circular RNAs (circRNAs) are characterized by their covalently closed, circular shape and resulting lack of 5′ and 3′ ends. Consequently, circRNAs are resistant to exonucleases that target the ends of RNA strands, causing circRNAs to be very stable, with a half-life of approximately 48 h [5] compared to the 10 h lifespan of messenger RNA (mRNA) [6]. Due to their stability, circRNAs are often enriched in exosomes and peripheral blood plasma [7].

MicroRNAs (miRNAs) are small noncoding RNAs that play a vital role in gene expression regulation, with developmental, maintenance and pathogenic roles. In conjunction with effector proteins, miRNAs form a miRNA-induced silencing complex [8] that targets complementary sequences of mRNAs, inhibiting their translation or inducing their degradation. Furthermore, circRNAs have been found to be miRNA “sponges” in that their sequences may contain binding sites for complementary seed sequences of miRNAs. Through binding to these miRNAs, circRNAs significantly affect mRNA stability and expression [9].

circRNA–miRNA interactions have been shown to contribute to the pathology of neurodegenerative disorders and cancers. For example, Xu et al. [10] showed that overexpression of the circRNA *circNFIX* in glioma tumor samples reduces expression of the miRNA *miR-34a-5p*, which targets *NOTCH1*, consequently promoting glioma progression through the Notch signaling pathway. In healthy individuals, miRNA-7 binds the circRNA *ciRS-7*, allowing *UBE2A* to coordinate clearance of amyloid peptides. However, in sporadic Alzheimer’s disease, there is a deficit in *ciRS-7* resulting in increased expression of *miRNA-7* and subsequent downregulation of *UBE2A* [11]. This causes amyloid accumulation and formation of plaque deposits in the brain, contributing to the molecular pathology of Alzheimer’s disease. Moreover, Sang et al. [12] reported downregulation of a particular circRNA known to be coexpressed with *SNCA* during pramipexole treatment in Parkinson’s disease patients. Downregulation of this circRNA results in upregulation of its sequestered miRNAs, causing a reduction in apoptosis and promoting autophagy.

Here, we investigate perturbation of the circular transcriptome in ALS, interactions between significantly differentially expressed (DE) circRNAs and ALS-associated miRNAs, and consequently the circRNA–miRNA–mRNA network.

## 2. Results

### 2.1. Differentially Expressed circRNAs

The circRNAs that were detected by both CIRI2 and CIRCexplorer2 were determined to be bona fide and therefore utilized in downstream analyses. Statistical analysis determined that the median variance explained by any of the variables in any of the tissues tested was <4.5% (Table S1). These variables were thus not included in the differential expression testing procedure.

There were 1.26 to 1.83 times more circRNAs detected in ALS samples than in control samples across all tissue types, yet 75% to 90% of circRNAs detected in control samples were also detected in ALS samples (Table 1). Moreover, 2- to 10-fold more circRNAs were detected in ALS samples than in control samples; hence, more circRNAs in ALS samples were specific to the condition and tissue types.

t-SNE plots of circRNA isoforms showed clustering among samples, which indicates that these circRNAs have unique expression profiles in each tissue that do not fully differentiate between ALS and healthy controls (Figure S1a,c,e).

Expression (in CPM) of all bona fide circRNAs was examined to find the average expression value for each tissue and condition; this value often fell below 100 CPM, but some circRNAs were expressed >10,000 CPM. Interestingly, spinal cord thoracic (SCT) exhibited a difference in circRNA expression distribution patterns compared to spinal cord cervical (SCC) and spinal cord lumbar (SCL), with higher expression in control samples than ALS samples.

Analysis of circRNA differential expression indicated 20, 2 and 70 DE circRNAs with adjusted *p* values < 0.05 in SCC, SCL and SCT, respectively. With respect to SCC and SCT, there were 5.7-fold and 1.8-fold more upregulated circRNAs than downregulated circRNAs. SCL only expressed two DE circRNAs, both of which were downregulated. There were no overlaps in DE circRNAs between the three tissues.

### 2.2. Binding Capacity of miRNAs to Differentially Expressed circRNAs

miRNA binding site detection analysis was carried out on the DE circRNAs (adjusted *p* values < 0.05) from SCC and SCT tissues. To reduce false positives, all miRNAs utilized were chosen if they were found to have binding sites in at least ten DE circRNAs; 914 miRNAs for each tissue fit this criterion. Overall, the average number of miRNA binding sites per circRNA was 6.5 and 6.15 for tissues SCC and SCT, respectively.

**Figure 1:**
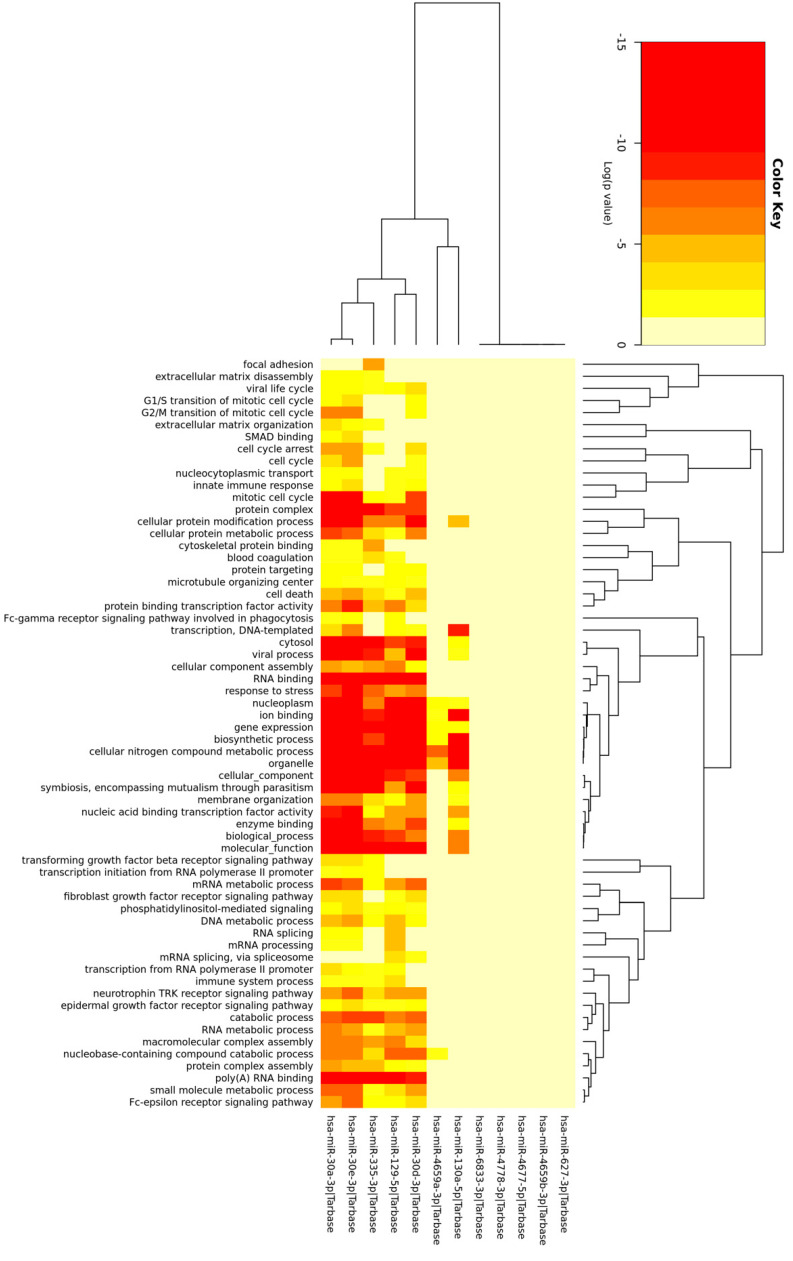
Heatmap of GO terms of the mRNA targets of 12 miRNAs. Heatmap showing which GO terms are associated with each miRNA and the colors associated with the *p* value for the miRNA/GO term pair.

The mRNA targets of the miRNAs with the highest number of binding sites in upregulated circRNAs across both tissues were utilized in gene ontology (GO) enrichment analysis. GO terms associated with ‘RNA binding’, ‘cellular protein processes’ and ‘cellular components’ were the most prominent among the genes targeted by this subset of miRNAs (Figure 1).

### 2.3. Comparison of Differentially Expressed circRNAs and mRNAs

Linear RNA expression analysis was performed to investigate perturbation of the linear transcriptome in ALS, which was then compared to DE circRNAs and miRNA binding site patterns. In total, 3556 DE genes were detected, and Table 2 displays the number of these genes with adjusted *p* values < 0.05 with respect to their tissue type.

All tissues showed more upregulated than downregulated DE genes. Interestingly, SCC harbored the most DE genes, comprising 84% of the 3556 nonredundant DE genes and indicating a greater degree of condition-specific linear RNA transcriptomic profile variation in the cervical region of the spinal cord.

t-SNE plots of linear RNA isoforms indicated that linear RNAs have unique expression profiles in each tissue that are able to differentiate relatively well between ALS and healthy controls with regard to SCC and SCT tissues but less so for SCL (Figure S1b,d,f).

DE linear RNAs were predominantly expressed at values <5 TPM, a trend that was consistent across conditions and spinal cord regions.

**Figure 2:**
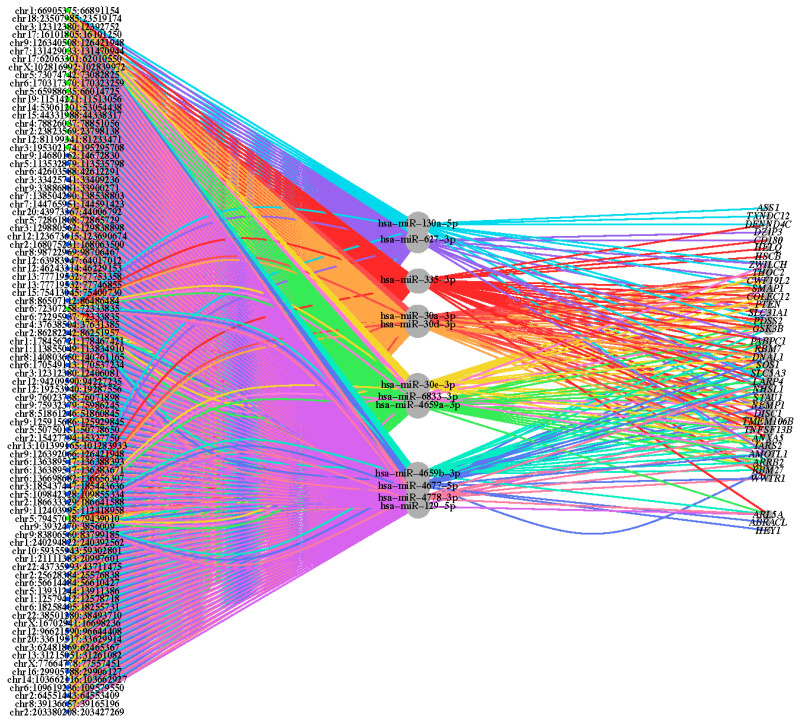
Network of circRNA–miRNA–mRNA interactions. A tripartite network graph displaying DE circRNAs (**left**), targeted miRNAs (**middle**) and targeted mRNAs (**right**), and the interactions between species. The node size equates to the log_2_ transformation of the number of interactions associated with each node, and the interaction line color relates to the associated miRNA. The green-colored circRNAs originated from SCC tissue, and the blue-colored circRNAs originated from SCT tissue.

To assess the ability of upregulated circRNAs to sequester specific miRNAs resulting in derepression of miRNA-targeted mRNAs, correlations among the three types of molecules were analyzed. mRNA targets of the miRNAs utilized in the GO analysis were evaluated for differential expression and log_2_(fold change) status. There were 37 genes found to be both miRNA targets and DE, suggesting perturbed mRNA expression in ALS as a result of increased miRNA sequestering by upregulated circRNAs in ALS. This analysis revealed a circRNA–miRNA–mRNA interaction network involved in ALS (Figure 2).

DE gene expression of the 37 genes identified in the circRNA-miRNA-mRNA network, shown in Figure S2, reveals that all genes are more highly expressed in the ALS SCC and SCT cohorts compared to their respective control cohorts. This corroborates the hypothesized circRNA-miRNA-mRNA networks proposed in this study whereby circRNAs are sequestering candidate miRNAs which would otherwise function to control and suppress mRNA targets.

GO enrichment analysis was performed on the 37 genes to assess the biological function of genes affected by circRNA–miRNA interactions in ALS disease (Table 3). The most enriched GO term in this analysis was ‘GO:1901699: Cellular response to nitrogen compound’, with a *p* value of 2.97 × 10^−6^. Other enriched GO terms relate to positive regulation of synaptic transmission, cell‒cell signaling and cellular response to hormone stimuli.

## 3. Discussion

circRNAs are involved in the pathogenesis of many diseases and have potential as biomarkers. In particular, circRNAs have been shown to interact with miRNAs in brain cancers and some neurodegenerative disorders, which has an effect on mRNA target expression and consequently on disease outcomes [10,11,12]. This study aimed to investigate circRNA–miRNA–mRNA interactions in the ALS disease profile and reports novel findings, enhancing our current understanding of ALS.

### 3.1. ALS-Specific Circular Transcriptome

To our knowledge, this body of work is the first to describe circular transcriptome perturbance in spinal cord tissues in ALS. circRNAs have been identified as potential biomarkers of ALS disease in peripheral blood [13], with 274 upregulated and 151 downregulated DE circRNAs, similar in proportion to our differential expression results (62 upregulated and 30 downregulated DE circRNAs). There are, however, key differences between the two studies. We analyzed differential expression of circRNAs in spinal cord tissue areas where ALS pathology originates and performed spatial circRNA expression analysis, and we provide evidence for functional circRNA–miRNA–mRNA interactions in ALS.

In this study, 92 circRNAs (nonredundant and adjusted *p* values < 0.05) were found to be DE in ALS samples. Variable numbers of DE circRNAs across each spinal cord region were detected, and none occurred in more than one region. This shows that circRNA expression is highly spatially regulated within each part of the spinal cord in a disease-specific manner. Integration of circRNA expression data with patient clinical data may reveal additional value as prognostic biomarkers if certain circRNA profiles can be correlated with the clinical features of the disease. Some clinical information is available for the NYGC dataset; however, analysis of these data was outside the scope of this project. This will certainly be investigated in future studies.

### 3.2. circRNA‒miRNA‒mRNA Interactions

The miRNA sponging effect is the best described function of circRNAs, and there has been sustained interest in understanding the impact on epigenetic regulation processes. This study aimed to determine miRNAs potentially ALS-associated and have binding sites in upregulated circRNAs. The target mRNAs of selected miRNAs were predicted using the TargetScanHuman database (version 6.0) [14,15,16,17] and correlated with the differential expression of upregulated mRNAs in the tissues of interest in ALS patients. An inference was made regarding derepression of certain mRNA transcripts in response to competition of miRNA binding due to upregulation of circRNAs that bind miRNAs.

The miRNAs selected in this analysis were determined to contain a large number of binding sites for upregulated DE circRNAs. After ensuring these miRNAs to be biologically relevant in circRNA and mRNA comparative analysis, they were assessed for GO enrichment of biological processes, and many of the enriched GO terms are associated with ALS pathways. The ‘RNA binding’ GO category is ALS related, as numerous causative genetic mutations in RNA-binding proteins have been detected in ALS patients [18]. Furthermore, many studies have shown that dysfunction of RNA metabolism and cytoplasmic mislocalization of RNA-binding proteins may contribute to progression of ALS [18]. Another significantly enriched GO category was ‘nucleoplasm’, which may be associated with ALS pathology in that dysfunction in nucleocytoplasmic transport and the consequent mislocalization of TDP-43 may contribute to ALS and other neurodegenerative diseases [19]. Furthermore, FUS is a component of the nucleoplasm but becomes mislocalized to the cytoplasm when certain ALS-causing mutations are present [20]. Other GO categories that are linked to ALS include ‘protein complex’ [21], ‘mitotic cell cycle’ [22] and ‘TRK receptor signaling’ [23]. Thus, the miRNAs selected for this analysis were regarded as biologically relevant through their association with canonical ALS cytopathology and through their effect on mRNA targets.

For further analysis, potential miRNA target genes were identified and extracted from linear RNA DE analysis if they were found to be significantly differentially expressed and upregulated in the disease condition. This comparison revealed 55 genes that fit these criteria. To further examine the relevance of these genes to the ALS disease profile, they were subjected to GO analysis utilizing the TarBase database [24], which indicated a high incidence of ALS GO terms such as ‘GO:0198738: cell‒cell signaling by wnt’ and ‘GO:0032436: positive regulation of proteasomal ubiquitin-dependent protein catabolic process’. Wnt signaling has been shown to be involved in the pathogenesis of ALS, as neurodegeneration upregulates expression of Wnt2 and Wnt7a, which activate Wnt signaling [25]. Additionally, the ubiquitin‒proteasome system may be disrupted in ALS, leading to studies assessing therapeutic strategies for neurodegenerative diseases and cancer [26,27,28].

The present study provides indications for a complex circRNA–miRNA–mRNA interaction network in ALS. However, further validation studies analyzing differential expression of the highlighted miRNAs and specific interactions between molecules within the different areas of the transcriptome are essential. It would also be valuable to explore the correlation between the number of binding sites among the upregulated circRNAs and the actual degree of consequent derepression of cognate linear RNA transcripts to fully confirm this mechanism as the primary modality for upregulation of the mRNAs we identified in this study. Overall, this research reveals a potentially unexplored aspect of ALS pathogenesis that demands further study. The insight provided by such studies will aid in the development of new therapeutic strategies for ALS.

In conclusion, our report presents, for the first-time, insights into differential circRNA expression in three distinct regions of the spinal cord of ALS patients. The circular RNA–microRNA–mRNA interactions analysis suggests modulation of mRNA expression as a result of miRNA sequestration by differentially expressed circRNAs. Together, circRNAs, specifically expressed in ALS spinal cord tissue, present a novel target for development of molecular diagnosis strategies for this pathology.

## 4. Materials and Methods

All relevant software and scripts utilized the GENCODE comprehensive gene annotation file (version 33) [29] and UCSC reference genome (GRCh38/hg38) package. This research includes computations using the computational cluster Katana supported by Research Technology Services at UNSW Sydney [30].

### 4.1. Dataset Structure and Quality Control

RNA sequencing (RNA-seq) data were retrieved from New York Genome Centre (NYGC) from deceased patients diagnosed with ALS and deceased patients classified as ‘nonneurological controls’. The FASTQ data files utilized in this study originate from three tissue types: spinal cord cervical (SCC), spinal cord thoracic (SCT) and spinal cord lumbar (SCL). The distribution between the number of ALS-affected and healthy patient samples was approximately 2.6:1 (Table 4). Tissue samples were in some cases collected from the same patient; the samples originated from 49 patients in total, with 4 patients providing 3 tissue samples and 12 providing 2 tissue samples. NYGC sequenced the samples using Illumina HiSeq and Ribo-Zero RNA depletion in the library preparation of all samples.

The main limitations with regard to the reliability of this project were the RNA integrity numbers (RINs)—an assessment of RNA quality—and the postmortem intervals (PMIs)—the number of hours between the patient’s death and sample collection. The ALS samples utilized in this study had RINs of ^3^7, and most were collected less than 15 h after death. The control dataset consisted of samples with RIN values of 3.5–7.3, and the majority of samples were collected within 25 h of death. To determine the effect PMI and RIN had on the ensuing analyses, circRNAs were filtered using the edgeR (version 3.32.1) [31] ‘filterByExpr’ function such that only circRNAs with at least 10 counts per million mapped reads (CPM) in at least nine samples were selected; nine samples refer to 70% of the size of the smallest tissue-type dataset. Read counts were transformed using the variance stabilizing transformation ‘vst’ function in the DESeq2 package (version 1.30.1) [32]. We assessed the variance explained by RIN, sex, group and PMI, where group represented ALS or control, using mixed effects linear models, as implemented in the variancePartition package (version 1.20.0) [33]. We fitted mixed effects linear models with the following formula: ~RIN + PMI + (1|sex) + (1|group).

All raw RNA-seq data files were trimmed using Trimmomatic (version 0.38) [34] to remove poor-quality sequences and technical sequences such as adapters.

To evaluate consistency in read quality and mapping rate, the raw and trimmed reads were run through FastQC (version 0.11.8) [35], STAR (version 2.7.2b) [36] and HISAT2 (version 2.1.0) [37].

### 4.2. circRNA Detection and Differential Expression Analysis

circRNA detection was carried out using two workflow pathways, and the outputs converged to determine which circRNAs were consistently detected using both methods. This dual-tool approach is considered imperative in reducing false positives, and the circRNAs detected by both tools are more likely to be bona fide. The first method consisted of aligning the trimmed reads to the reference genome using the BWA-MEM algorithm (version 0.7.17) [38]. The outputted SAM-formatted files were then entered into CIRI (version 2.0.6; CIRI2) [39], producing a list of circRNA candidates. The second method involved the trimmed reads being mapped to the reference genome using STAR (version 2.7.2b) [36] with the ‘--sjdbOverhang’ flag specified. Next, the STAR output was run through CIRCexplorer2′s (version 2.3.0) [40] parse and annotate pipelines, producing the second list of circRNA candidates. The CIRCexplorer2 output was filtered to select candidate circRNAs having two or more reads spanning the back-spliced junction. The circRNAs detected by both CIRI2 and CIRCexplorer2 were determined using inbuilt R functions (version 3.6.1) [41].

circRNA candidates, in the form of circRNA IDs and averaged read counts from CIRCexplorer2 and CIRI2 output, were run through R’s differential expression software packages limma (version 3.46.0) [42] and edgeR (version 3.30.0) [31]. The ‘trimmed mean of M values’ (TMM) normalization method was utilized.

### 4.3. Linear RNA Detection and Differential Expression Analysis

Linear RNA analysis was conducted using the Pertea et al. [43] protocol, in which HISAT2 (version 2.1.0) [37] and StringTie (version 1.3.4) [44] were employed for alignment and abundance estimation, respectively. As novel unannotated transcripts were not of interest in this study, the shortened protocol for linear RNA analysis was utilized. Alignment was conducted with reference annotation input followed by StringTie’s estimated abundance (e) module with the same reference annotation utilized for circRNA detection, which provides the relative abundance of genes and transcripts. The gene abundance flag (–A) option of StringTie provided a table of complete gene expression, including the expression values of all transcripts pertaining to a particular gene; the individual transcript expression values are otherwise found in the default StringTie.gtf output file.

Differential expression of genes and transcripts was analyzed using R’s limma [42] and edgeR [31] packages.

### 4.4. circRNA‒miRNA‒mRNA Interaction Analysis

miRNA binding sites within each significantly DE circRNA (adjusted *p* values < 0.05) sequence were identified using the circRNAprofiler R package (version 1.0.0) [45], in particular the ‘getMiRsites’ function. Only circRNAs from SCC and SCT samples were utilized for this analysis, as the SCL samples had very few circRNAs that met the *p* value criteria. The miRNA IDs used in this analysis were acquired from miRBase, all with a high confidence rating. This was achieved using the R packages scrapeR (version 0.1.6) [46], miRBaseConverter (version 1.14.0) [47] and stringr (version 1.4.0) [48].

The 12 miRNAs with the highest frequency of binding sites among upregulated circRNAs across both tissues were input into the mirPath online tool (version 3.0) [49] to determine associated GO terms. The TarBase database [24] option was utilized for all miRNA‒mRNA target predictions.

Using the same 12 miRNAs, we identified conserved miRNA targets and obtained context ++ scores associated with each miRNA‒mRNA target pair by utilizing relevant TargetScanHuman (version 6.0) [14,15,16,17] Perl scripts. These scripts output the mRNA “Gene ID” as RefSeq mRNA IDs, and hence, it was necessary to convert these IDs to HNGC gene symbols and Ensembl gene IDs using the R package biomaRt [50,51]. Upregulated DE genes from the linear RNA differential expression analysis with adjusted *p* values < 0.05 were then compared with the previously mentioned list of miRNA-targeted genes to determine genes that occurred in both lists and consequently may be affected by DE circRNAs via miRNA sponging. Comparative gene expression analysis of these upregulated DE genes was performed using CPM values for ALS and control cohorts in a tissue-specific manner. Differential expression significance (adjusted *p*-value) was determined using the pipeline described above in ‘Linear RNA detection and Differential Expression Analysis’. These potentially affected genes were input into Molecular Signatures Database [52,53,54] to obtain a set of GO terms associated with the gene set.

### 4.5. Data Visualization

t-SNE plots were constructed using the Bioconductor R packages ‘Rtsne’ [55] and ‘edgeR’ [31]. Default parameters were used, except that the perplexity setting was adjusted to values from 1.7 to 5.0 to improve the resolution of distance and the shape of clusters. Comparative gene expression plots were created in Prism v9.4.1 as box-and-whisker plots (min-max).

## Figures and Tables

**Table 1:** Average number of circRNAs detected in different sample types in different tissues.

Tissue	Condition	Number of Samples	Number of circRNAs Detected	Number of Detected circRNAs Common to ALS and Control Samples	Number of circRNAs Unique to the Condition
Spinal Cord Cervical	ALS	18	24,259	12,557	11,702
	Control	7	15,210		2653
Spinal Cord Lumbar	ALS	16	24,020	14,044	9976
	Control	6	18,262		4218
Spinal Cord Thoracic	ALS	16	20,581	12,100	8481
	Control	6	16,209		4109

**Table 2:** Number of DE genes in each tissue type with adjusted *p* values < 0.05.

		Spinal Cord Cervical	Spinal Cord Lumbar	Spinal Cord Thoracic
**Number of DE genes** **(adjusted *p* value < 0.05)**	**Upregulated**	1944	524	242
	**Downregulated**	1052	460	90
	**Total**	2996	984	332
**Total genes tested**		60,662	60,662	60,662

**Table 3:** Enriched GO terms from GO analysis of the miRNA-targeted mRNA gene set. The number of genes (targets of miRNAs sequestered by circRNAs) that correspond to each GO term and the enriched *p* value are listed.

GO Accession and Name	Number of Genes in Overlap	*p* Value
GO:1901699: Cellular response to nitrogen compound	7	2.97 × 10^−6^
GO:0198738: Cell‒cell signaling by wnt	6	6.94 × 10^−6^
GO:0050806: Positive regulation of synaptic transmission	4	9.41 × 10^−6^
GO:1901701: Cellular response to oxygen-containing compound	8	9.67 × 10^−6^
GO:0071375: Cellular response to peptide hormone stimulus	5	1.16 × 10^−5^
GO:0001837: Epithelial to mesenchymal transition	4	1.20 × 10^−5^
GO:0051100: Negative regulation of binding	4	1.39 × 10^−5^
GO:1905114: cell surface receptor signaling pathway involved in cell‒cell signaling	6	1.88 × 10^−5^
GO:0032870: Cellular response to hormone stimulus	6	1.98 × 10^−5^
GO:1901653: Cellular response to peptide	5	2.83 × 10^−5^

**Table 4:** Number of spinal cord biological samples utilized in ALS and control conditions.

Tissue	No. of Samples in ALS Cohort	No. of Samples in Control Cohort
Spinal Cord Cervical	18	7
Spinal Cord Thoracic	16	6
Spinal Cord Lumbar	16	6

## Data Availability

Specifications for all detected DE circRNAs are available at https://github.com/genieus/circRNA-NYG-ALS/blob/main/DE_circRNAs.txt. Raw sequencing data are available from NYGC on an application basis.

## Supplementary Materials

The following supporting information can be downloaded at: https://www.mdpi.com/article/10.3390/ijms232314665/s1.
