# Supplementary material for: Circular RNA Expression and Interaction Patterns Are Perturbed in Amyotrophic Lateral Sclerosis
Source: Int J Mol Sci. 2022 Nov 24;23(23):14665. doi: 10.3390/ijms232314665 (PMC9739162; doi:10.3390/ijms232314665)
Supplement: Supplementary file 1 [file ijms-23-14665-s001.zip › ijms-1962977-supplementary.pdf]

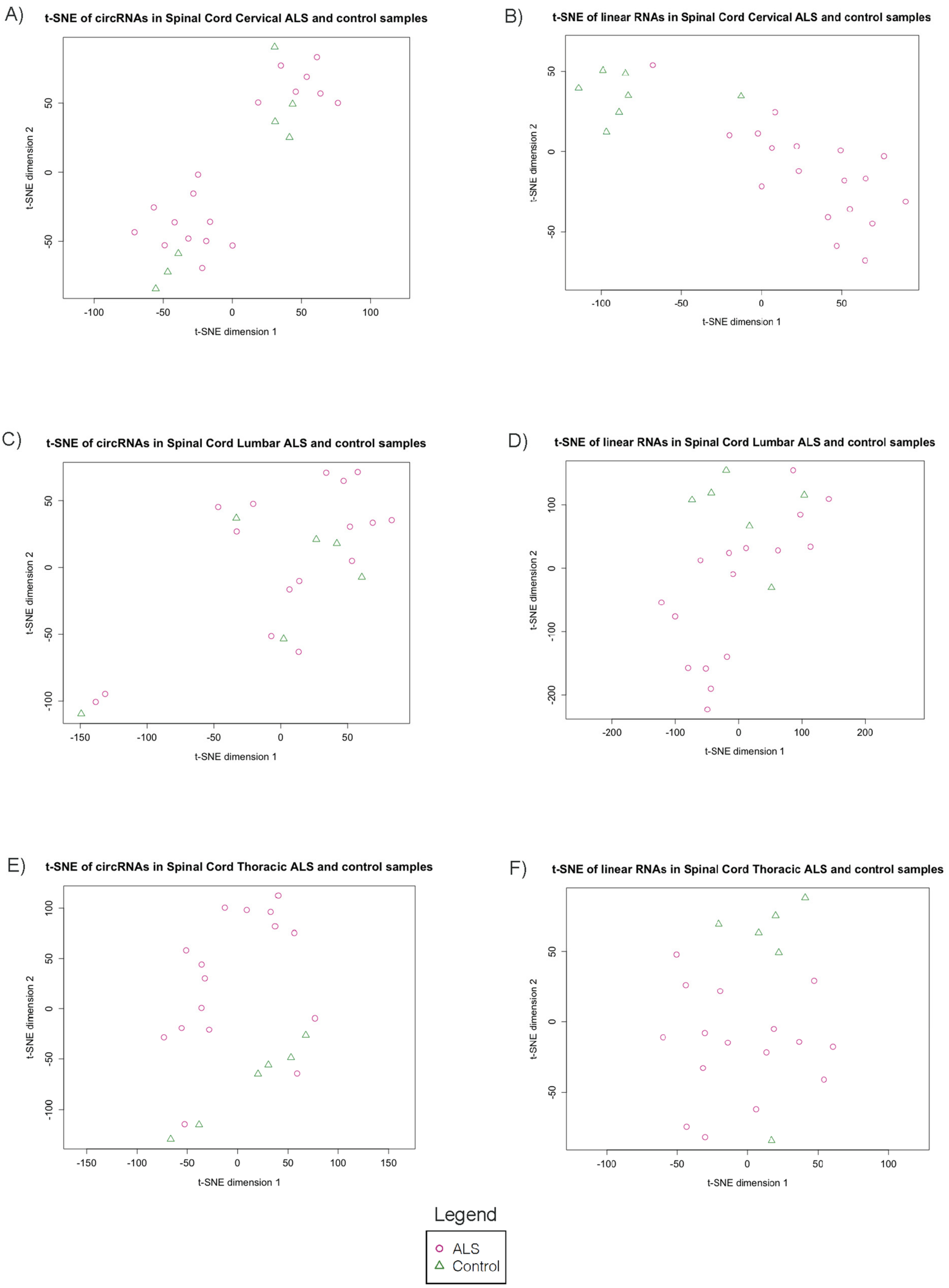

Figure S1. t-SNE plots of the circular transcriptome (A,C,E) and linear transcriptome (B,D,F). Distinct clusters can be seen in both sets of plots, and control and ALS samples appear in clusters together. However, in some plots, the control samples are closer together within a general area. The clustering within ALS samples indicates the variability of the disease.

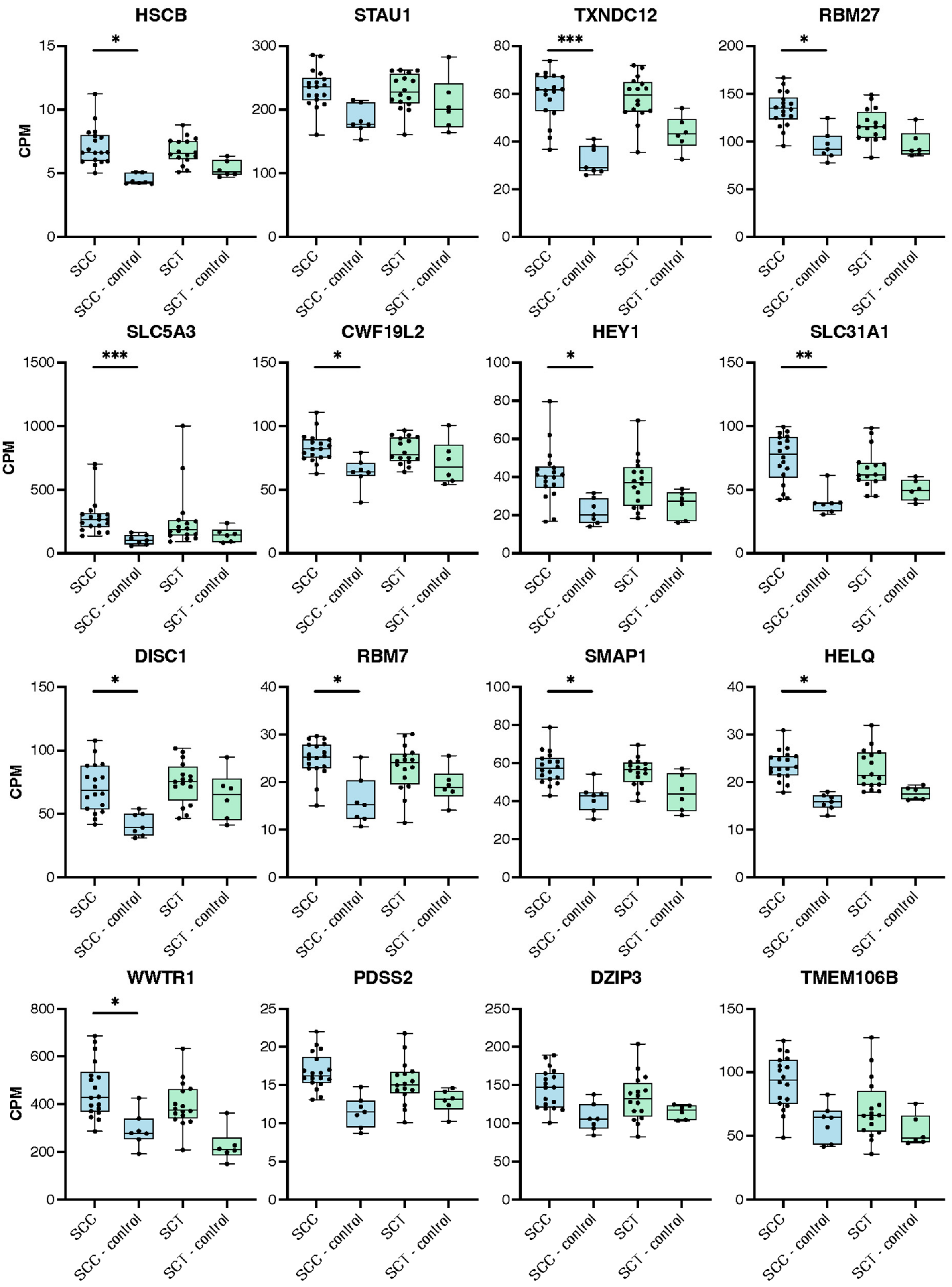
HSCB
STAU1
TXNDC12
RBM27
SLC5A3
CWF19L2
HEY1
SLC31A1
DISC1
RBM7
SMAP1
HELQ
WWTR1
PDSS2
DZIP3
TMEM106B
CPM
SCC
SCC - control
SCT
SCT - control
*
**
***

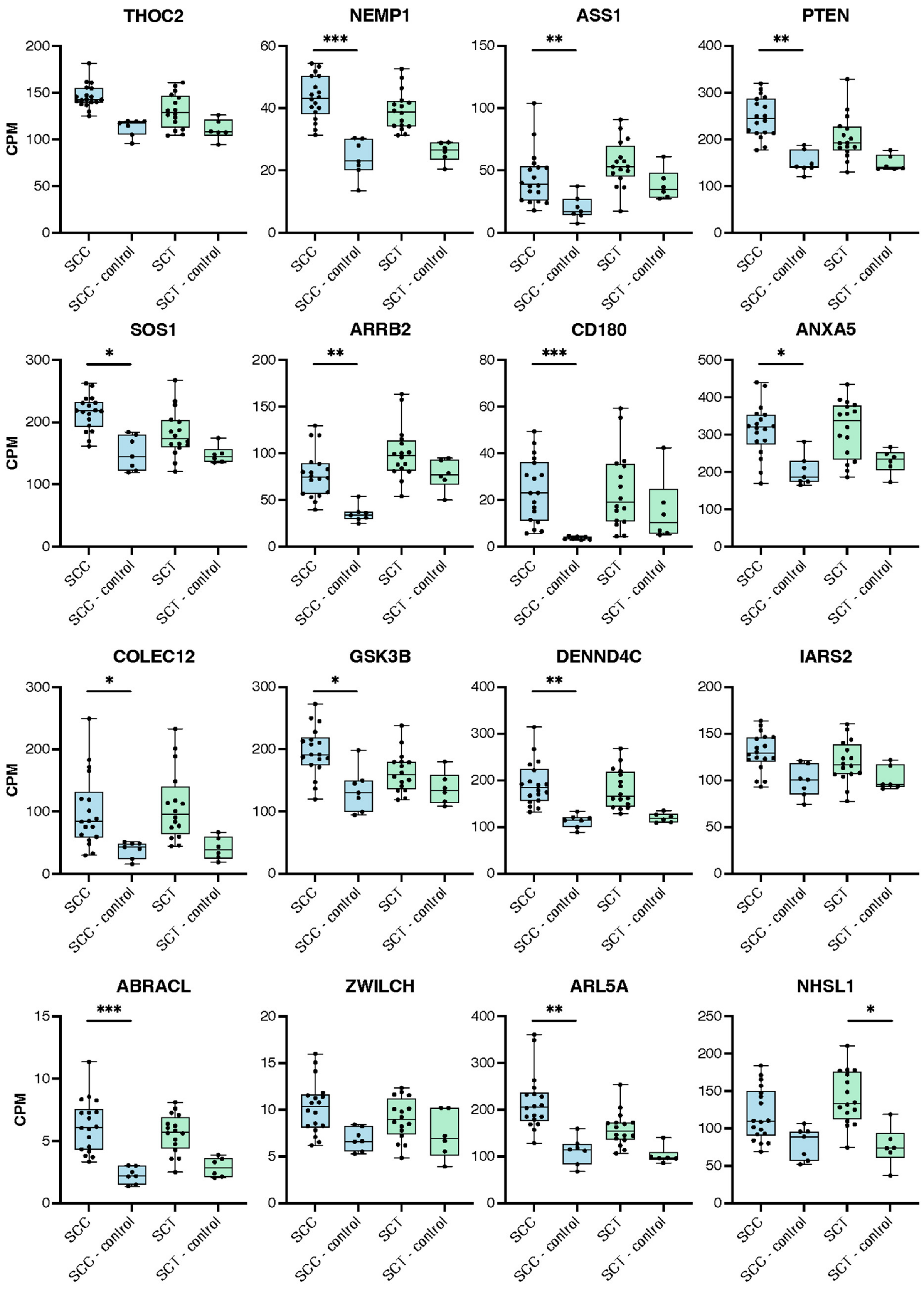
THOC2
NEMP1
ASS1
PTEN
SOS1
ARRB2
CD180
ANXA5
COLEC12
GSK3B
DENND4C
IARS2
ABRACL
ZWILCH
ARL5A
NHSL1
CPM
SCC
SCC - control
SCT
SCT - control
*
**
***

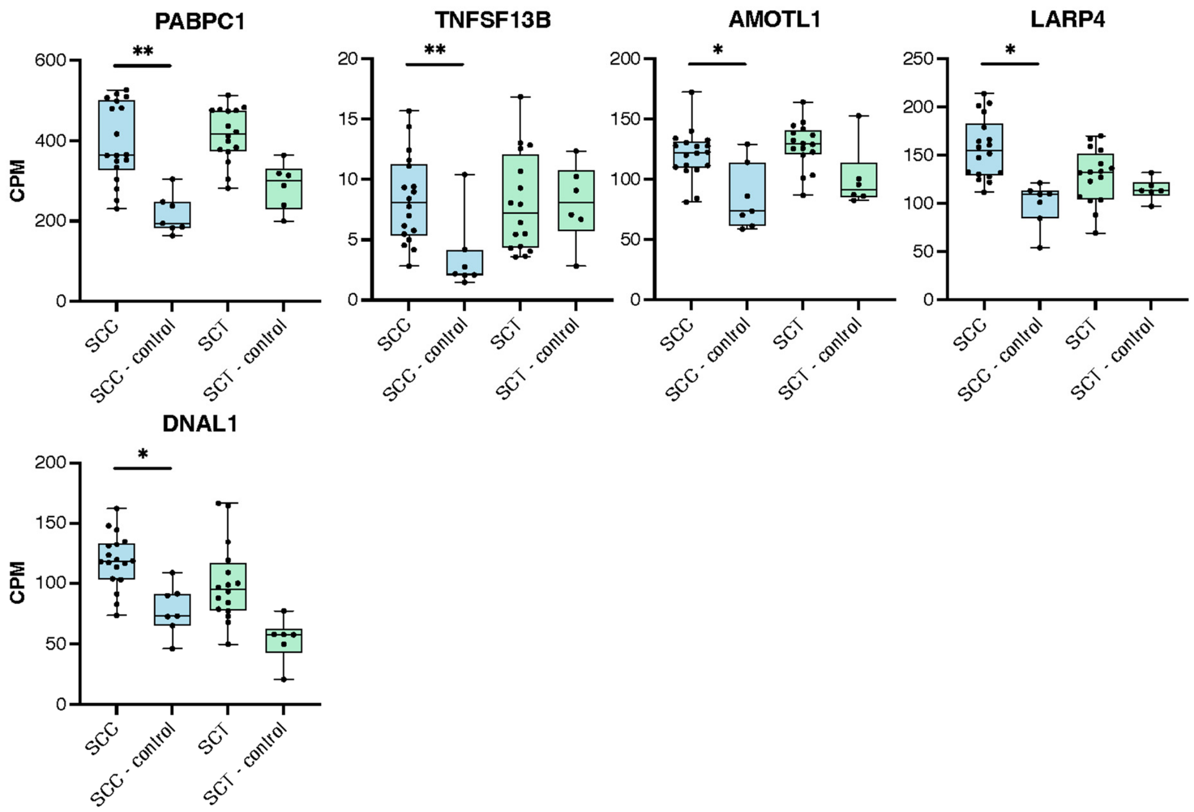

Figure S2. Differential expression of genes identified in the circRNA-miRNA-mRNA interaction network in SCC and SCT. Differential gene expression in SCC and SCT samples in ALS and control cohorts indicates lower expression in control cohorts for all genes identified in this interaction network and included in Figure 2. Upregulation of candidate gene expression in the ALS samples is suggestive of the hypothesized circRNA sequestering of candidate miRNAs which would otherwise be involved in genetic regulation as shown in the control samples. Blue: SCC; Green: SCT; * < 0.05, ** < 0.01, *** < 0.001.

Table S1. Median proportion of variance explained across all genes by each variable.

Tissue	Group	Sex	RIN	PMI	Residuals
SCC	0	0	0.035	0.018	0.89
SCL	0	0	0.026	0.022	0.906
SCT	0	0	0.044	0.021	0.885
